# The Interlinkages Between Ambient Temperature and Air Pollution in Exacerbating Childhood Asthma: A Time Series Study in Cape Town, South Africa †

**DOI:** 10.3390/children12121634

**Published:** 2025-12-01

**Authors:** Tshepo Kingsley Phakisi, Edda Weimann, Hanna-Andrea Rother

**Affiliations:** 1 Division of Environmental Health, School of Public Health, University of Cape Town, Anzio Rd, Observatory 7925, South Africa; tshepophakisi@hotmail.com; 2 Digital Health, Department of Information Systems, School of IT, Commerce Faculty, University of Cape Town, Rondebosch, Cape Town 7701, South Africa; edda.weimann@uct.ac.za; 3 TUM School of Medicine and Health, Children Campus Munich Schwabing, Technical University of Munich (TUM), 80804 Munich, Germany

**Keywords:** childhood asthma, climate change, temperature, air pollution, low- and middle-income countries, early warning systems

## Abstract

**Background:** Given the rapid global increase in asthma cases, understanding the impact of climate change on respiratory health is necessary for evidence-based policymaking, particularly in low- and middle-income countries (LMICs). Objectives: To estimate the short-term associations between temperature (mean and diurnal range), particulate matter (PM_2.5_ and PM_10_), nitrogen dioxide (NO_2_), ozone (O_3_), and childhood asthma exacerbations in Cape Town, South Africa. **Methods:** We analysed daily hospital records (*n* = 7753; 2009, 2014, 2019) alongside citywide air quality and meteorological data using negative binomial mixed-effects models and distributed lag non-linear models to capture delayed effects. **Results:** NO_2_ and PM_10_ were consistently associated with a higher exacerbation risk, with additional delayed effects observed for PM_2.5_, PM_10_, and NO_2_. Mean temperature and diurnal temperature range were also linked to an increased risk at short (lag 0–1) and medium (lag 4–5) delays. **Conclusions:** Temperature variability and traffic-related air pollution contribute to childhood asthma exacerbations in urban LMIC settings. The findings support child-centred early warning systems and stricter air quality controls aligned with WHO guidance.

## 1. Introduction

Climate change and air pollution are converging threats to child health, altering the exposures that trigger and exacerbate asthma in urban settings [1,2]. Asthma continues to be one of the most common chronic respiratory diseases affecting children globally, with its occurrence steadily increasing. Children are uniquely susceptible to environmental health hazards because of their developing lungs, higher ventilation rates per body weight, and the greater time they spend outdoors. These vulnerabilities make asthma outcomes particularly sensitive to changes in climate and air quality, leading to more severe and prolonged effects in children [1].

Among environmental risk factors, air pollution is consistently associated with the onset and worsening of asthma in children. Exposure to traffic-related pollutants, such as nitrogen dioxide (NO_2_) and particulate matter (PM_2.5_), has been estimated to contribute substantially to the burden of childhood asthma [3,4]. Exposure to NO_2_ is associated with about 1.85 million new paediatric asthma cases annually, and PM_2.5_ increases the risk of developing childhood asthma by 5%, with disproportionate burdens in dense, lower-income cities [3,4]. Additionally, pollutants such as particulate matter (PM_10_) and ozone (O_3_) are well-known respiratory tract irritants that can cause airway inflammation and increase the risk of asthma-related hospitalisation [4,5].

In South Africa, the prevalence of childhood asthma has increased drastically over recent decades, with nearly 20% of children aged 13–14 years affected [6]. This rapid rise cannot be explained solely by genetic factors or known environmental hazards, such as pollen, seasonality, and air pollution [7]. Recent studies suggest that climate variability may be an important additional driver, but it remains understudied in LMICs [8].

Extreme temperatures, whether hot or cold, along with diurnal temperatures, have been associated with worsening conditions through mechanisms such as airway inflammation, increased vulnerability to viruses, and changes in aeroallergen behaviour [8,9]. This suggests that climate-related temperature changes may interact with air pollution exposure to increase the risk in children.

Analyses specific to LMICs that jointly evaluate temperature and multiple pollutants for childhood asthma are scarce [7]. In South Africa, there is limited evidence on how temperature extremes and air pollution interact to influence asthma exacerbations in children [10,11]. Most studies have focused on air pollution and respiratory diseases in adults compared to children [12,13,14,15]. Olaniyan et al. (2020) studied air pollution and pollen exposure in children with asthma in the Western Cape, South Africa. The study also included weather variables but only used one year of data and focused on PM_2.5_ and NO_2_ [16].

Therefore, the present study examined the role of multiple air pollutants (NO_2_, PM_10_, PM_2.5_, and O_3_) and ambient temperature in influencing childhood asthma exacerbations in Cape Town, South Africa. The rapidly urbanising context and diverse pollutant sources make Cape Town a policy-relevant setting for studying these interlinkages. We analysed childhood hospital presentation data from 2009, 2014, and 2019, which corresponded to key milestones in the implementation of the South African National Ambient Air Quality Standards. We applied negative binomial mixed-effects and distributed lag non-linear models to quantify the immediate and delayed associations with asthma exacerbations. This study provides LMIC-specific evidence that complements the global paediatric literature and directly informs child-centred air quality alerts and climate adaptation planning in South Africa. The work bridges global paediatric evidence with LMIC policymaking by quantifying the temperature–pollution–asthma interlinkages in a major African city and highlighting actionable levers for child health protection.

## 2. Materials and Methods

### 2.1. Study Design

We used a time-series regression design to assess the effects of daily air pollution and temperature on childhood asthma exacerbations at the Red Cross War Memorial Children’s Hospital (RXH) in Cape Town, South Africa. Hospital records were linked to meteorological and air quality data from the South African Weather Services and the City of Cape Town.

### 2.2. Childhood Asthma Data

Child asthma data were collected from RXH, a tertiary referral hospital serving Klipfontein and Mitchells Plain, Rosebank, Mowbray, Rondebosch, and Salt River suburbs. Cape Town was the only South African location in Phase 1 of the International Study of Asthma and Allergies in Childhood (ISAAC), making it a suitable site for this study [17]. RXH is one of only two dedicated tertiary care children’s hospitals in the country [18]. We included inpatient and outpatient children who received asthma treatment at RXH in 2009, 2014, and 2019. Using a multiple cross-sectional design, we analysed health data at three distinct time points, with 2014 as the midpoint, providing a ten-year snapshot of data collected five years before and after.

We defined inpatients as children admitted for asthma treatment and outpatients as children treated for asthma without hospital admission. Asthma diagnoses were classified using the International Classification of Diseases, 10th Revision (ICD-10 Code), with codes J45.0 to J45.998. Data was extracted from CliniCom^TM^, a Patient Master Index system used in the Western Cape province. The variables collected included patient names, ages, addresses, ethnicity, gender, treatment date, ICD-10 Codes, and diagnosis. We reviewed the data of 15,811 patients during the study period. Our inclusion criteria were patients aged six–13 years who resided within the CCT at the time of treatment. As a result, 7753 patient visits to RXH (49.0%) met the study criteria, thus excluding 8058 patient treatments (51.0%) due to age or non-CCT residency status. Among the included patients, most were outpatients, with only 409 (5.3%) treated for asthma as inpatients.

### 2.3. Air Quality and Temperature Data

**Figure 1:**
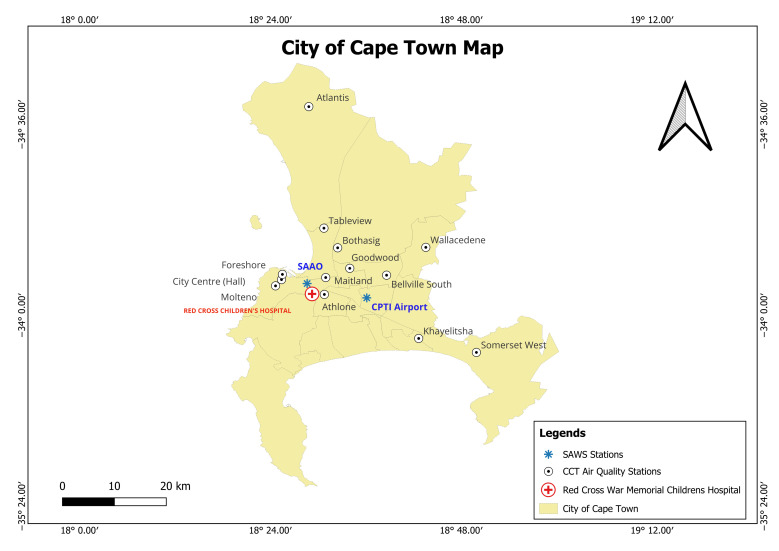
City of Cape Town Air Quality Monitoring Stations.

We collected air quality data for PM_2.5_, PM_10_, NO_2_, and O_3_ and temperature data (i.e., daily maximum and minimum) for our time series analysis. We used three cross-sectional analyses for the study years 2009, 2014, and 2019, covering two equal five-year intervals. These years corresponded to key national air quality and health policy milestones [19,20,21]. In 2009, South African National Ambient Air Quality Standards (NAAQS) were established. By 2014, the transition to a more stringent NAAQS, particularly for PM10, had occurred. 2014 was also a midpoint between 2009 and 2019, enabling an evenly spaced interval for trend analysis. Additionally, in 2019, the CCT began monitoring ambient PM_2.5_ in September 2017 [15,22,23,24]. We collected daily air quality data from 14 CCT air quality monitoring stations (Figure 1). Four air pollutants, ozone (O_3_ µg/m^3^), nitrogen dioxide (NO_2_ µg/m^3^), and particulate matter ≤10 (PM_10_ µg/m^3^) and ≤2.5 (PM_2.5_ µg/m^3^), were selected based on their effects on asthma [25,26]. Before calculating the daily averages for the pollutants, we collected hourly pollutant concentration values from the 11 monitoring stations during the study period (Table S1). Due to variability in data completeness across monitoring stations and days, we computed a citywide hourly average for each pollutant by averaging the available concentration values across all stations for each hour. This approach not only helped fill data gaps by leveraging measurements from other monitoring stations for the same date and time but also provided an estimate of citywide exposure.

By averaging the hourly pollutant concentrations across various stations, we assumed that residents of the city were exposed to similar air quality. A similar approach has been used in air pollution and health time series studies conducted in Cape Town [12,14,15,27,28]. We then calculated daily 24 h average concentrations for PM_2.5_, PM_10_, NO_2_, and O_3_ from these citywide hourly averages, requiring at least 18 1 h valid measurement values per day according to the ISO 17025 guidelines [29]. Similar calculations were used in Cape Town [12,14,15,27,28]. We did not perform data imputation for the days when no station recorded data; these were left as missing.

Daily temperature data for 2009, 2014, and 2019 were obtained from the SAWS for the city. We collected data for a full calendar year (January to December) from two monitoring sites within the city: Cape Town International Airport and the South African Astronomical Observatory (SAAO) (Figure 1). The dataset included both the minimum and maximum daily temperatures. The consolidated daily maximum temperature was calculated by averaging the daily maximum temperatures recorded at both the sites. Similarly, the consolidated daily minimum temperature was calculated by averaging the daily minimum temperatures at both locations. The daily average temperature was calculated by averaging the maximum and minimum temperatures of the day. Diurnal temperature was defined as the variation between the daily maximum and minimum temperatures of the day. It was calculated as the difference between the consolidated daily maximum and minimum temperatures for each day during the study period.

Air quality and temperature datasets were used to explore their relationship with childhood asthma treatment cases at the hospital using time-series regression analysis within a multi-cross-sectional design.

### 2.4. Statistical Analysis

A descriptive statistical analysis was conducted to summarise the demographic characteristics of the study participants and environmental exposure levels for each study year (2009, 2014, and 2019). We calculated frequencies and percentages for categorical variables, including type of admission, sex, age group, area of residence, type of asthma, and season. We compared the distributions across years using the chi-square test. For continuous environmental variables (average temperature, DTR, PM_2.5_, PM_10_, NO_2_, and O_3_), the mean and standard deviation were calculated, and differences between years were assessed using a one-way analysis of variance (ANOVA). Where data were only available for two years, an independent sample *t*-test was performed, except for PM_2.5_, which had only one year of data. The statistical significance was set at *p* < 0.05. We employed a snapshot approach focusing on three distinct years (2009, 2014, and 2019) as these years were linked with important milestones in South Africa and the CCT air quality policy [26]. This approach enabled us to explore the relationship between daily environmental exposures (temperature and air pollutants) and childhood asthma exacerbations without aggregating data over extended periods.

A monotonic relationship was explored among asthma exacerbation cases, daily temperature, and air quality variables using Spearman’s rank correlation coefficients. A non-parametric method suitable for assessing pairwise associations that may not be linear was used. This helped to detect potential collinearity among environmental exposures, which are significant risk factors for asthma exacerbation.

A negative binomial mixed-effects regression model was used to model asthma exacerbations. This was an appropriate model for the over dispersed count data of daily hospital asthma treatment visits. Monitoring stations were treated as random effects to account for spatial variability in exposure, and the year was added as a second random effect in the multi-year models.

Bivariate and multivariable analyses were conducted on the exposure variables of temperature (average and DTR) and air pollution (PM_2.5_, PM_10_, NO_2_, and O_3_). These variables were considered fixed effects, and monitoring stations were treated as random effects. We analysed these models separately for 2009, 2014, and 2019, depending on data availability. A pooled model was used for all three years of the study. This included exposure variables with consistently available data on PM_10_ and the temperature. We also conducted another pooled model for NO_2_ and O_3_ across the two years (2014 and 2019). For these models, we used monitoring stations and years as the random effects.

In the multivariable model for each year, we adjusted for season and included interaction terms between average temperature and season, with the average temperature centred to improve interpretability. However, diurnal temperature was excluded from the final models because of nonsignificant results and poor model fit, as indicated by the Akaike Information Criterion (AIC) and Bayesian Information Criterion (BIC) values.

In the final model for 2019, where complete data were available, including PM_2.5_, we applied a distributed lag non-linear model (DLNM) with lags 0–7 days to assess the immediate and delayed short-term effects of air pollutants and temperature on asthma exacerbations. This lag structure is commonly used in time-series studies on asthma, air pollution, and temperature [12,30,31,32,33].

These models provided estimates of the Incident Rate Ratios (IRRs) with 95% confidence intervals (CI), representing the relative change in asthma cases associated with each unit change in exposure concentration values [34]. We assessed the model fit using the AIC and BIC to determine the best model and avoid overfitting the data. For the lagged negative binomial mixed effects models, we examined the residual diagnostic plots, which included QQ plots and residuals versus the predicted values. We also tested for overdispersion and zero inflation across all lags (0–7). However, no evidence of overdispersion was found, as *p*-values ranged from 0.18 to 0.33, and no evidence of zero-inflation was found, with *p*-values between 0.79 and 0.88. Overall, across all lags, the models had an appropriate fit for analysing the association between air pollution, temperature, and childhood asthma hospital treatment visits. We did not perform data imputation for missing variables to minimise the researchers’ influence on the dataset. All the statistical analyses were conducted in R (version 4.3.1). The fitted models are expressed as follows:

Main Model: Negative binomial mixed-effects multivariable model (lagged)

$$\begin{array}{cc} log\left(E\left[N\;hosp\;asthma\right]\right) & \\ & =\beta 0+\beta 1\;\times \;{PM}_{2.5}\;\left(lag\;k\right)+\beta 2\;\times \;{PM}_{2.5}\;\left(lag\;k\right)+\beta 3\\ & \times \;{NO}_{2}\;(lag\;k)+\beta 4\;\times \;O_{3}\;(lag\;k)+\beta 5\;\times \;Temp\;(lag\;k)+\;\beta 6\\ & \times \;Season_{winter}+\beta 7\;\times \;Season_{autumn}+\beta 8\;\times \;Season_{spring}+\beta 9\\ & \times \;(Temp\;(lag\;k)\times Season_{winter})+\beta 10\\ & \times \;(Temp\;(lag\;k)\times Season_{autumn})+\beta 11\\ & \times \;(Temp\;(lag\;k)\times Season_{spring})+station \end{array}$$

In general, for these models, N hosp asthma represents the number of daily observed asthma treatment hospital visits, β0 is the intercept coefficient for the model (constant), β1, β2, β3, β4, β5, β6, β7, β8, β9, β10, β11 and is the regression coefficient representing our fixed effects, including air pollutants, temperature, seasons, and the interaction between temperature and seasons, as indicated in the models. For the lagged model, lag k represents the days before the current day in the model. The station represents our random effects single-year models, and in the multi-year models, we included station + year as random effects.

## 3. Results

### 3.1. Demographic and Health Characteristics of Study Participants

Table 1 outlines the sociodemographic and clinical characteristics of children treated for asthma at the RXH in 2009, 2014, and 2019. Across the three study years, a total of 7753 asthma treatment visits were recorded as follows: 2009 (N = 1953), 2014 (N = 2701), and 2019 (N = 3099). We observed a 10% increase in asthma treatment visits between 2009 and 2014 and a further 5% increase between 2014 and 2019. Most asthma cases were outpatients (>94%), predominantly male (56–58%), and aged 6–9 years (62–64%). The distribution of area of residence and asthma type differed significantly between the years (*p* < 0.000001), with higher increases in Athlone (50–64.5%) and allergic asthma (36–61%). The seasonal distribution of asthma cases has remained consistent over the years. In winter, the number of cases was consistently the highest compared to other seasons, closely followed by that in summer.

### 3.2. Temperature Variables and Air Pollutants

Table 2 outlines the summary statistics for the environmental variables across the three study years. There was a consistent decrease in the concentration of PM_10_ from 36.19 µg/m^3^ (SD 17.10) in 2009 to 21.08 µg/m^3^ (SD 8.96) in 2019. O_3_ concentrations increased from 20.77 µg/m^3^ (SD 16.57) in 2014 to 41.16 µg/m^3^ (SD 11.47). NO_2_ showed a decreased concentration from 19.62 µg/m^3^ (SD 6.21) in 2014 to 12.86 µg/m^3^ (SD 7.36) in 2019. For PM_2.5_, we had data only for 2019. For 2009, we had missing data for O_3_, NO_2_, and PM_2.5_. Both temperature variables (average and DTR) remained constant throughout the three-year study period. These environmental variables (temperature and air pollution) differed significantly between years, except for PM_2.5_, which was excluded from the test.

### 3.3. Association Between Childhood Asthma, Air Pollution, and Temperature Variables

**Figure 2:**
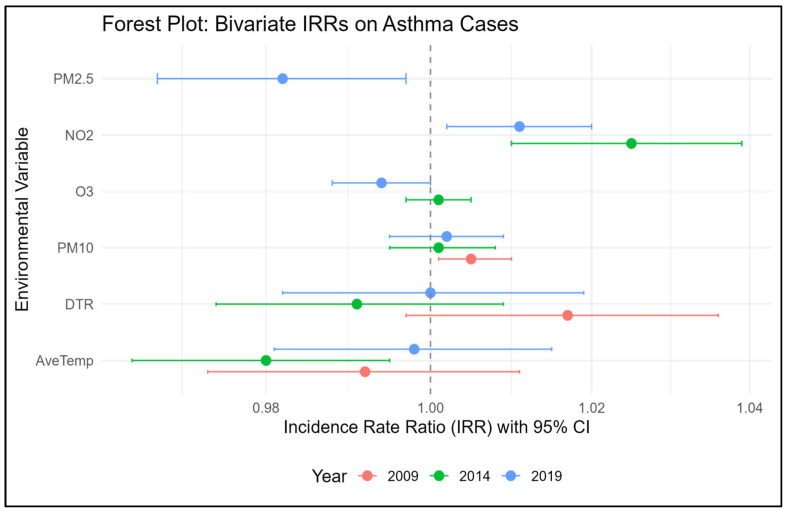
Forest Plot of Bivariate analysis: asthma exacerbation risk associated with environmental variables stratified by year.

Figure 2 presents a forest plot of the estimates of the bivariate regression analysis that explored the relationship between childhood asthma and environmental exposure (temperature and air pollution). We found that NO_2_ exposure was consistently associated with an increased risk of childhood asthma in 2014 and 2019. In 2014, NO_2_ had a significant positive association with asthma (IRR 1.025, 95% CI: 1.011–1.039), indicating a 2.5% increase in daily asthma cases per unit increase in NO_2_. However, in 2019, NO_2_ had a significant positive association with asthma (IRR 1.011, 95% CI: 1.002–1.020), indicating a 1.1% increase in daily asthma cases per unit increase in NO_2_.

For PM_10_, the pollutant was positively associated with asthma but was only significant for 2009 (IRR 1.005, 95% CI: 1.001–1.009), indicating a 0.5% increase in asthma cases per unit increase in PM_10_ concentration. O_3_ and PM_2.5_ had a significant negative association with asthma cases in 2019; however, in 2014, O_3_ had a positive nonsignificant association. Over the three years, the average temperature had a negative association with asthma cases, with only 2014 being statistically significant. (Figure S1) shows the correlations between asthma and environmental variables in 2009, 2014, and 2019. The most significant correlations between asthma and environmental variables were PM_10_ in 2009 (r = 0.04, *p* < 0.01) and NO_2_ in 2014 (r = 0.09, *p* < 0.001) and 2019 (r = 0.04, *p* < 0.01). We observed similar significant positive associations for PM_10_ and NO_2_ with asthma exacerbations in our multi-year analysis (Figure S2).

### 3.4. Multivariable Delayed Association Between Childhood Asthma, Air Pollution, and Temperature Variables

**Figure 3:**
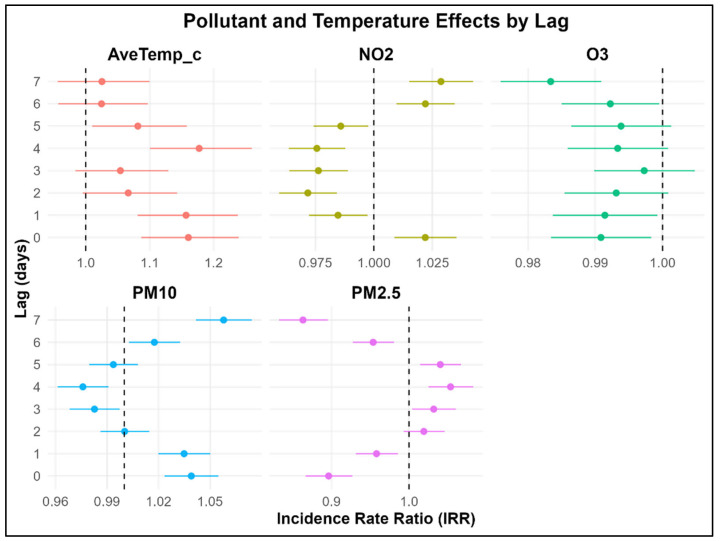
Association between temperature, air pollutants, and childhood asthma: multivariable multi-pollution lagged model for 2019.

Figure 3 shows the relationship between air pollutants, temperature, and asthma exacerbations in 2019. NO_2_, PM_10_, PM_2.5_, and mean average temperatures were all significant predictors of asthma exacerbations across different lags while controlling for seasons. Distributed lag analysis showed time-varying associations between exposure and asthma exacerbation. For PM_2.5_, significant decreases in risk were observed at lag 0 (IRR 0.90, 95% CI: 0.87–0.93), lag 1 (IRR 0.96, 95% CI: 0.93–0.99), lag 6 (IRR 0.95, 95% CI: 0.93–0.98), and lag 7 (IRR 0.86, 95% CI: 0.83–0.90), while there was an observed significant increased asthma risk at lag 3–5 with the peak at lag 4 (IRR 1.05, 95% CI: 1.03–1.08). This indicates a delayed response of a 5% increase in asthma exacerbations for lag 4 for each unit increase in PM_2.5_.

PM_10_ showed increased asthma risk at lag 0 (IRR 1.04, 95% CI: 1.02–1.06), lag 1 (IRR 1.04, 95% CI: 1.02–1.05), lag 6 (IRR 1.01, 95% CI: 1.003–1.03), and lag 7 (IRR 1.05, 95% CI: 1.04–1.07), while a reduced asthma risk was observed at lag 3–4. These results indicate that for every unit increase in PM_10_ at lags 0, 1, 6, and 7, asthma exacerbations increased by 1–5%. For NO_2_, a mixed effect was observed with increased risks in lag 0 (IRR 1.02, 95% CI: 1.008–1.03, *p* < 0.001), lag 6 (IRR 1.02, 95% CI: 1.009–1.03, *p* < 0.001), and lag 7 (IRR = 1.02, 95% CI: 1.01–1.04) and a reduced risk at lag 1–5. These results indicate that for each unit increase in NO_2_ at lags 0, 6, and 7, the incidence of asthma exacerbation increased by 2%. For O_3_, consistent with our earlier analysis, there was a significant negative relationship in the earlier lags, but an overall negative relationship throughout the lags. Overall, O_3_ had a slight inverse effect on childhood asthma.

Temperature showed a strong significant positive association at lag 0 (IRR 1.16, 95% CI: 1.08–1.23), lag 1 (IRR 1.15, 95% CI: 1.08–1.23), and lag 4 (IRR 1.17, 95% CI: 1.10–1.25). (Figure S3) shows the effects of (a) seasonality and (b) its interaction with temperature on the incidence of childhood hospitalisation. Except for summer across all years, Autumn, Winter and Spring amplified the risk of asthma exacerbations for lag 0–5 (IRR > 1.5) with a reduced temperature effect. However, for the interaction between temperature and seasonality, in summer, a unit increase higher than the average daily temperature increased the risk of childhood asthma exacerbations, whereas the effect was reduced in other seasons.

## 4. Discussion

This is the first South African study to show that ambient temperature and air pollution increase the risk of childhood asthma hospitalisation. Our study showed that higher than average daily temperatures and one unit increase in air pollutants (PM_10_ and NO_2_) levels were strongly associated with same-day childhood asthma-related hospitalisations. Our lagged analysis showed delayed associations between temperature and air pollutants (PM_2.5_, PM_10_, NO_2_) with asthma exacerbations in children aged six–13 years, suggesting that multiple biological pathways may be involved. In contrast, we observed a significant inverse association with O_3_. Seasonality also emerged as an independent predictor of childhood asthma, separate from the effects of temperature and air pollution. Compared to summer, all other seasons were strongly associated with an increased risk of childhood asthma. However, in summer, higher-than-average temperatures were associated with an increased risk of childhood asthma compared to other seasons.

Overall, this study highlights the increased risk of asthma exacerbations in children from LMICs due to the combined effects of temperature and air pollution. These findings align with those of several global studies, indicating that air pollution and temperature significantly affect childhood asthma exacerbations [35,36,37,38,39,40].

Since 2009, the number of childhood asthma cases treated at RXH has increased, consistent with the current literature on the global rise in childhood asthma, particularly in LMICs [7,41]. However, this study could not provide population-based rates because it focused solely on cases treated at RXH rather than the broader population. Among the children treated for asthma at RXH, 56.9% were male. Although the number of females treated was lower, their admission rate was higher (54.5%). Similar sex-based patterns have been reported in other studies [42,43,44]. Boys generally have smaller airway diameters, increased allergic inflammation, and higher serum Immunoglobulin E (IgE) levels, which may contribute to the higher prevalence of asthma in childhood compared to girls [44,45]. While some studies reported no sex differences in hospitalisation for asthma exacerbations, [46], others found that boys are twice as likely as girls to be hospitalised with no sex disparity in childhood asthma hospitalisation due to exacerbations [45]. It is worth noting that the higher incidence of asthma in males tends to switch to females during puberty [45]. These findings suggest that sex is a significant risk factor for childhood asthma exacerbations, with effects that vary by age. Additionally, the interaction between climate change, age, and sex adds further complexity. Studies have reported that younger boys are more susceptible to heat-related asthma, whereas older boys are more sensitive to cold temperatures than girls [9,47].

Recent studies showed seasonal influences on asthma exacerbations [32,48]. This study found that childhood asthma in Cape Town occurred more often in autumn, winter, and spring than in the summer. These seasonal trends may be due to pollen exposure, indoor and ambient air pollution due to combustion-related sources, including traffic emissions and residential wood burning, as well as dust exposure. In Cape Town, tree pollen peaks in spring, while grass pollen is from spring through summer, with higher concentrations in coastal areas [49]. Fungal spores peak in autumn and spring, contributing to asthma exacerbations [49,50]. Cape Town’s wet winters is associated with increased viral respiratory infections, which may worsen asthma [51,52]. Children spend more time indoors during winter, increasing exposure to household allergens and pollution [11,53]. Similar seasonal trends in Taiwan showed a higher asthma prevalence in winter than in summer [53]. While temperature extremes, particularly heat, are important predictors of exacerbations, this study found that heat outside the summer season had a lesser effect on exacerbation. The findings highlight how the environment and childhood behaviour impact seasonal asthma risk in Cape Town.

Furthermore, our analysis showed temperature may modify the effect of air pollution on childhood asthma hospitalisation with immediate effects (lag 0) and delayed inflammatory response at lag 4, both consistent with biological plausibility of public health relevance [54]. This study’s correlation analyses showed significant covariation between temperature and air pollutant levels, highlighting a challenge in isolating their effects on health. Global research on temperature as an effect modifier of air pollutants is growing, including in South Africa and Cape Town [55] where studies reported stronger effect of PM_10_ and NO_2_ on cardiovascular and respiratory admissions during warm days [15,31,56]. In this study, higher-than-average temperatures were associated with increased risk of childhood asthma hospitalisation by about 15–17% for multiple lags (lag 0–4), with a narrow CI, indicative of significant public and clinical health relevance. Furthermore, higher temperature was correlated with increased childhood asthma and showed positive associations with PM_10_, PM_2.5_, and NO_2_. Similar findings were reported in Australia, where DTR was correlated with PM_10_, O_3_, NO_2_,and childhood asthma exacerbations [33]. These patterns suggest that hotter days and greater temperature fluctuations coincided with higher pollutant levels in Cape Town.

DNLM analysis identified PM_2.5_ as a significant predictor of childhood asthma in Cape Town. Its small size allows for deep lung penetration and oxidative stress, triggering airway inflammation [36]. In winter and spring, PM_2.5_ levels in the city exceeded the WHO air quality guideline primarily due of traffic and biomass burning [21]. While PM_10_ and NO_2_ from traffic, industrial, residential, and harbour-related activities unique to Cape Town were consistently associated with childhood asthma [57]. Highest pollutant levels in winter and the lowest in summer, consistent with increased winter emissions and summer wind dispersal (the “Cape Doctor”) [21,57]. Children living near highways, traffic-congested areas, harbours, or densely populated areas are particularly vulnerable because of frequent outdoor exposure to high concentrations [16,57].

The distinct lag patterns for each pollutant provide insights into the mechanisms. PM_10_ immediate and late (lag 7) suggests both instant irritation and a delayed inflammatory response. PM_2.5_ at lag 4, suggest deep lung penetration and a systemic inflammatory response [36], while the complex lag pattern for NO_2_ showed both harmful and inverse associations, reflecting its role as a marker for traffic-related pollution mixtures and confounding by co-emitted pollutants [58,59].

Interestingly, O_3_ showed a negative association with childhood asthma exacerbations, contrary to the current research [60,61]. This relationship is unlikely to represent the true protective effect. Our analysis of mean O_3_ concentrations were increased from 2014 (20.8 ± 16.6 µg/m^3^) to 2019 (41.2 ± 11.5 µg/m^3^), whereas NO_2_ concentrations decreased from 2014 (19.6 ± 6.2 µg/m^3^) to 2019 (12.9 ± 7.4 µg/m^3^). Our correlation of NO_2_ and O_3_ shifted from positive in 2014 to negative in 2019, consistent with the expected NOx titration effect in urban areas. This process may create an apparent inverse association between O_3_ and asthma exacerbations, which may mask the direct impact of NOx on childhood asthma exacerbations [62,63].

### Limitations and Strengths of the Study

Understanding the impact of air quality on child health requires reliable data on air pollution type, source, and concentration [64,65]. Our analysing the CCT’s monitoring and archiving systems showed limited good-quality data from a few stations suitable for analysis (Table S1). Previous reports have highlighted data inadequacy in the Western Cape, this is despite CCT following the United States Environmental Protection Agency (USEPA) and ISO/IEC 17025:2017standards with SANAS TR07-03 quality controls [22,23,66].

Due to limited data, we assumed all participants experienced similar pollution exposure across the city. In line with other time series studies in Cape Town, we used the citywide averaging to capture temporal fluctuations in pollution rather than spatial differences [15,16,17,18,31]. To reduce the potential bias, we allocated children to stations near their homes and used a mixed-effects analysis with a random intercept for stations to account for the differences across the city without regard for mobility. Our lack of buffer radius may have avoided excluding cases but potentially increased the risk of exposure misclassification [67]. Additionally, PM_2.5_ monitoring in Cape Town started in late 2017, thus limiting the analysis to 2019 exposures. Future studies with continuous PM_2.5_ measurements would better assess long-term links to childhood asthma.

Furthermore, missing data on humidity, viral infections, socioeconomic status, and second-hand smoke exposure may have introduced potential bias [68]. Overall, this study’s use of mixed-effects model helped account for the temporal and spatial variability in exposures and outcomes. This provided a robust framework for examining short-term temporal associations at the population level.

This study used routinely collected real-world air pollution, temperature, and health data. Although some air quality datasets were incomplete across periods and pollutants, the data provide valuable insights into relationships between temperature, air pollution, and childhood asthma exacerbations in Cape Town.

While our study highlights an association between temperature, air pollution, and childhood asthma, poor quality data in Africa remains a significant barrier. Future research should integrate personal monitoring devices, time-series models, and spatial analyses to better capture exposure dynamics [69]. Improved air quality monitoring and data accessibility are crucial for protecting vulnerable populations, particularly children, from the interlinked threats of air pollution and climate change.

## 5. Conclusions

This initial time-series ecological design in Cape Town, South Africa (2009–2019) found associations between childhood asthma exacerbations and temperature, PM_2.5_, PM_10_, and NO_2_. Although the confidence intervals were narrow, indicating more precise estimates, causal interpretations were limited. Thus, the results warrant future research with more complete exposure data. Seasonality showed significant association with childhood asthma, and temperature predicted PM_2.5_, PM_10_, and NO_2_ concentrations. This supports previous research linking temperature, air pollution, and asthma exacerbation in children. The Department of Health (DoH) and Department of Forestry, Fisheries, and Environment (DFFE) should address temperature risks through climate-change adaptation strategies. The DoH should ensure that municipalities and stakeholders, such as the Provincial DoH, SAWS, and other departments, implement the National Heat-Health Action Guidelines. The DFFE should enforce stricter compliance with the NAAQS, aligning with WHO air quality guidelines, and enhance public access to SAAQIS through environmental health literacy. The SAWS should improve temperature monitoring and collaborate with the Department of Education to establish early warning systems in schools to protect the students. Provinces must implement climate change adaptation plans that address children’s respiratory health.

More accurate data are needed to study children’s exposure to temperature and air pollutants. Long-term air pollutant data must be collected and made available in South Africa. Policymakers must improve the air quality in urban areas. This study emphasises the need to fulfil the Paris Agreement to avoid further temperature increases and protect the health of future generations.

## Figures and Tables

**Table 1:** Demographic characteristics of children with asthma treated at RXH in 2009, 2014, and 2019 (N = 7753).

Characteristics	2009 (N = 1953)	2014 (N = 2701)	2019 (N = 3099)	*p*-Value
	n (%)	n (%)	n (%)	
Type of admission				0.0822
Inpatient	95 (5)	129 (5)	185 (6)	
Outpatient	1858 (95)	2572 (95)	2914 (94)	
Gender				0.277
Male	1113 (57)	1506 (56)	1792 (58)	
Female	840 (43)	1195 (44)	1306 (42)	
Age				0.246
Group (6–9)	1211 (62)	1736 (64)	1943 (63)	
Group (10–13)	742 (38)	965 (36)	1156 (37)	
Area of residence				<0.000001
Athlone	1020 (52)	1352 (50)	1998 (64.5)	
Khayelitsha	607 (31)	896 (33)	654 (21.1)	
City Hall	89 (5)	126 (5)	100 (3.2)	
Other	237 (12)	327 (12)	347 (11.2)	
Types of asthma				<0.000001
Predominantly Allergic asthma	703 (36)	1635 (61)	1775 (57)	
Other Asthma	1250 (64)	1066 (39)	1324 (43)	
Season of the year				0.383
Summer	537 (27)	707 (26)	845 (27)	
Autumn	521 (27)	677 (25)	774 (25)	
Winter	514 (26)	725 (27)	847 (27)	
Spring	381 (20)	592 (22)	633 (20)	

Seasons: summer (December, January, and February), autumn (March, April, and May), winter (June, July, and August) and spring (September, October, and November).

**Table 2:** Summary statistics for temperature and air pollutants in 2009, 2014, and 2019.

Environmental Exposures (Unit)	Mean (SD)	Mean (SD)	Mean (SD)	*p*-Value
	2009	2014	2019	
Average Temp (°C)	18.0 (3.8)	18.3 (3.9)	18.0 (3.5)	<0.0001 *
DTR (°C)	9.3 (3.7)	9.9 (3.6)	9.6 (3.4)	<0.0001 *
PM_10_ (µg/m^3^)	36.2 (17.1)	25.4 (10.0)	21.1 (9.0)	<0.0001 *
O_3_ (µg/m^3^)	-	20.8 (16.6)	41.2 (11.5)	<0.0001 #
NO_2_ (µg/m^3^)	-	19.6 (6.2)	12.9 (7.4)	<0.0001 #
PM_2.5_ (µg/m^3^)	-	-	(4.5)	-

(-) = data not available; type of test: (*) = ANOVA; (#) = *t*-test.

## Data Availability

The data presented in this study are available upon request from the corresponding author of this paper. However, owing to legal and privacy issues, these data are not publicly available.

## Supplementary Materials

The following supporting information can be downloaded at: https://www.mdpi.com/article/10.3390/children12121634/s1, Figure S1. Spearman’s correlation coefficients between daily air pollutants and temperature variables in the City of Cape Town for three study years 2009, 2014, and 2019. Figure S2. Forest Plot of Bivariate analysis: asthma exacerbation risk associated with environmental variables for multiple years. Figure S3. Seasonal and Temperature effect on childhood asthma cases at RXH. Table S1. Air quality data usability received from the City of Cape Town Air quality monitoring station.

## Abbreviations

The following abbreviations are used in this manuscript:

DLNM	Distributed Lag Non-Linear Model
DTR	Diurnal Temperature Ranges
IgE	Immunoglobulin E
CCT	City of Cape Town
GHGs	Greenhouse Gases
ICD-10 Code	International Classification of Diseases Codes
IQR	Interquartile Range
IRR	Incidence Rate Ratio
LMICs	Low- and middle-income countries
NAAQS	South African National Ambient Air Quality Standards
NO_2_ μg/m^3^	Nitrogen Dioxide
O_3_ μg/m^3^	Ozone
PM_10_ μg/m^3^	Particulate Matter with a diameter smaller than 10 micrograms
PM_2.5_ μg/m^3^	Particulate Matter 2.5
RXH	Red Cross War Memorial Children’s Hospital
SANAS	South African National Accreditation System
SD	Standard Deviations
