# Supplementary material for: The Interlinkages Between Ambient Temperature and Air Pollution in Exacerbating Childhood Asthma: A Time Series Study in Cape Town, South Africa
Source: Children (Basel). 2025 Dec 1;12(12):1634. doi: 10.3390/children12121634 (PMC12732075; doi:10.3390/children12121634)
Supplement: Supplementary file 1 [file children-12-01634-s001.zip › children-3953063-supplementary.pdf]

The interlinkages between ambient temperature and air pollution in exacerbating childhood asthma: A time series study in Cape Town, South Africa

Tshepo Kingsley Phakisi [1], Edda Weimann [2,3] and Hanna-Andrea Rother [1,*]

[1]Division of Environmental Health, School of Public Health, University of Cape Town, Anzio Rd, Observatory, 7925 South Africa. tshepophakisi@hotmail.com (TKP). andrea.rother@uct.ac.za (HAR).

[2]University of Cape Town, Commerce Faculty, Department of Information Systems, Digital Health, Rondebosch, 7701, South Africa. edda.weimann@uct.ac.za

[3]Technical University of Munich, School of Medicine & Health, Health Campus Schwabing, 80804 Munich, Germany

[*]Correspondence: andrea.rother@uct.ac.za; Tel.: +27-21-406-6721.

Supplementary material:

Ambient Air Quality Data Received from the City of Cape Town.

Table S1 presents information about the air quality data we received from the City of Cape Town. Some of the data received had gaps of over a month but the method of calculating city wide hourly

data across the same pollutant assisted us in having more complete hourly dataset prior to computing the citywide daily averages.

Table S1. Air quality data usability received from the City of Cape Town Air quality monitoring station.

Air quality monitoring station	O_3 ug/m^3			NO_2 ug/m^3			PM2.5 ug/m^3			PM10 ug/m^3		
	2009	2014	2019	2009	2014	2019	2009	2014	2019	2009	2014	2019
Atlantis	-	-	✓	-	×	-	-	-	-	-	-	-
Athlone	-	-	-	-	-	-	-	-	-	-	-	-
City Hall	-	-	-	-	✓	-	-	-	-	-	-	-
Plattekloof	-	×	×	-	×	×	-	-	-	-	×	✓
Goodwood	×	×	×	-	✓	-	-	-	-	✓	✓	×
Khayelitsha	-	-	-	-	×	-	-	-	✓	✓	×	×
Bothasig	-	-	-	-	✓	-	-	-	-	-	-	-
Tableview	-	-	-	-	✓	✓	-	-	✓	-	×	✓
Molteno	-	✓	×	-	-	-	-	-	-	-	-	-
Foreshore	-	-	-	-	-	-	-	-	×	-	×	×
Bellville	-	-	-	-	-	-	-	-	-	-	×	-
Summerset West	-	-	-	-	-	-	-	-	-	-	-	-
Wallacedene	-	-	×	-	-	-	-	-	-	-	×	-

Legends: × = Data received and not usable (no readings/too many gaps)
- = Data not received/not measured
✓= Data received and usable (there were gaps, but it was usable)

Bivariate analysis: Spearman's Correlation Coefficient Analysis

Figure S1 presents the monotonic relationship between asthma exacerbations, air pollutants and temperature variables in 2009, 2014, and 2019. The most significant correlations between asthma and environmental variables were PM10 in 2009 ($r = 0.04$, $p < 0.01$) and NO2 in 2014 ($r = 0.09$, $p < 0.001$) and 2019 ($r = 0.04$, $p < 0.01$). There was a strong positive correlation between DTR and PM10 in all years: 2009 ($r = 0.68$, $p<0.001$), 2014 ($r = 0.57$, $p<0.001$), and 2019 ($r = 0.55$, $p<0.001$). DTR was also positively correlated with NO2 in 2014 ($r = 0.47$, $p<0.001$) and in 2019 ($r = 0.60$, $p< 0.001$), including with PM2.5 ($r = 0.65$, $p< 0.001$), but was negatively correlated with O3 in 2019 ($r =-0.10$, $p< 0.001$).

A significant negative correlation was observed between O3 and PM2.5 in 2019 ($r = -0.38$, $p < 0.001$), and a significantly weaker negative correlation was observed between O3 and NO2 in 2019 ($r = -0.07$, $p < 0.001$), including a significant negative correlation with the average temperature in 2014 ($r = -0.25$, $p < 0.001$). The average temperature was positively correlated with PM10 across all years, and the strongest correlation was observed for PM2.5 in 2019 (r = 0.64, p

< 0.001). For PM10, we observed a strong positive correlation with NO2 in 2014 ($r = 0.37$, $p < 0.001$) and 2019 ($r = 0.38$, $p < 0.001$). In 2019, we also observed a very high correlation between PM10 and PM2.5 ($r = 0.790$, $p < 0.001$).

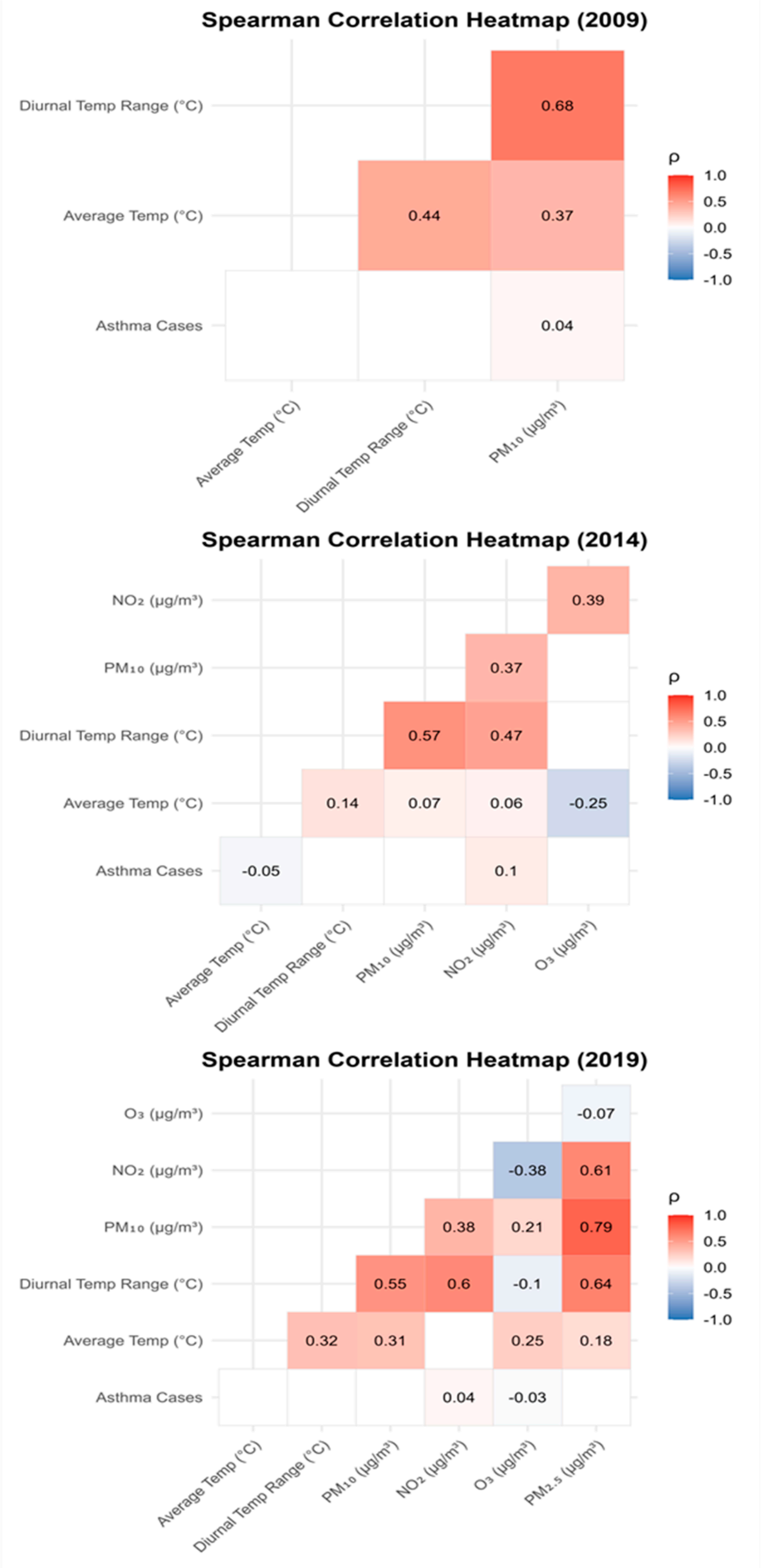

Figure S1. Spearman's correlation coefficients between daily air pollutants and temperature variables in the City of Cape Town for three study years 2009, 2014, and 2019.

Bivariate analysis: Negative Binomial Mixed Effect Regression Analysis

Multi-year single pollutant or temperature variable regression model for asthma exacerbations

Figure S2 presents a forest plot of the bivariate regression estimates examining the relationship between childhood asthma and environmental exposure (temperature and air pollution) across multiple years. Our analysis covered three years (2009, 2014, and 2019) for average temperature, DTR, and PM10, and two years (2014 and 2019) for NO2 and O3.

For the three-year period, PM10 was the only exposure variable with a statistically significant positive association with asthma cases (IRR 1.005:95% CI: 1.001 – 1.008, $p < 0.05$), indicating a 0.5% increase in asthma treatment visits per-unit increase in PM10 concentration.

Over the two years, NO2 was the only pollutant significantly associated with asthma (IRR 1.014:95% CI: 1.006 – 1.022, $p < 0.0001$), representing a 1.4 increase in asthma cases per unit increase in NO2 concentration.

Average temperature showed a significant negative association with asthma cases, suggesting a protective effect, whereas DTR and O3 were not significantly associated with asthma exacerbations.

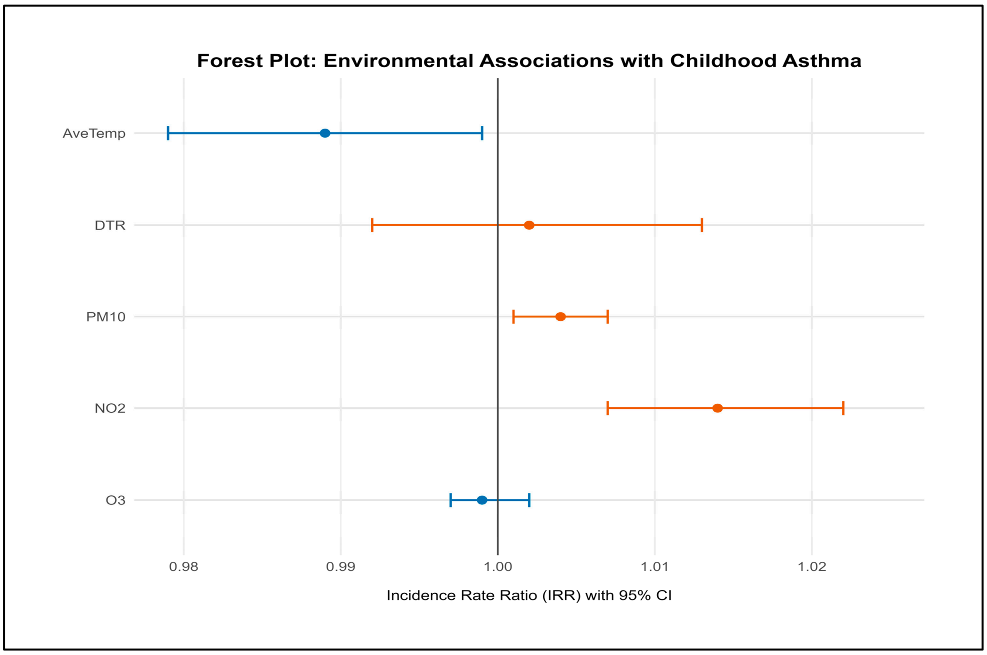

Figure S2. Forest Plot of Bivariate analysis: asthma exacerbation risk associated with environmental variables for multiple years.

Multivariable analysis: Negative Binomial Mixed Effect Regression Analysis

Figure S3 shows the effects of (a) seasonality and (b) its interaction with temperature on the incidence of childhood hospitalisation. Except for summer across all years, Autumn, Winter and Spring amplified the risk of asthma exacerbations for lag 0 – 5 (IRR > 1.5) with a reduced temperature effect. However, for the interaction between temperature and seasonality, in

summer, the unit increase higher than the average daily temperature increased the risk of childhood asthma exacerbations, whereas the effect was reduced in other seasons.

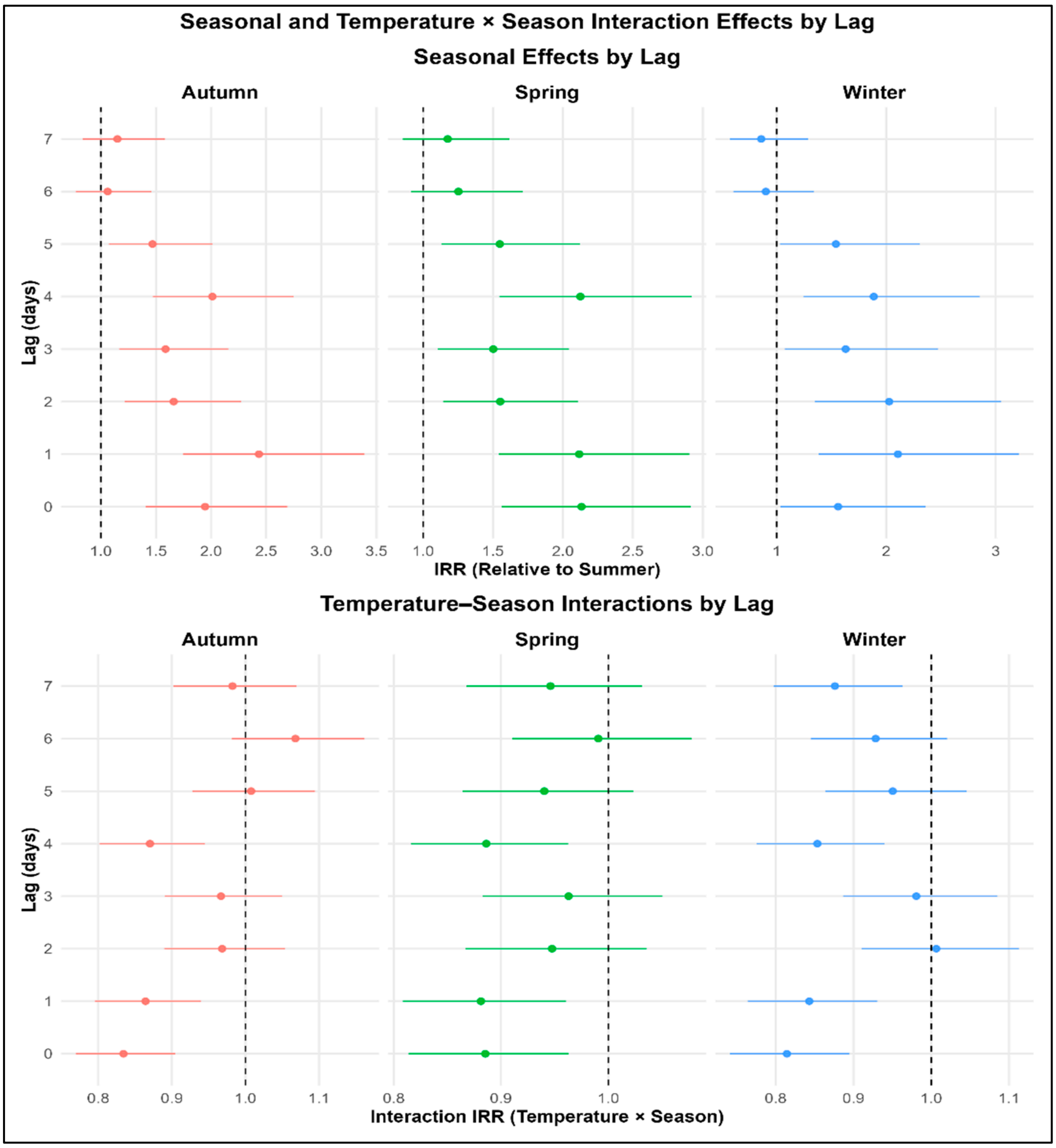

Figure S3. Seasonal and Temperature effect on childhood asthma cases at RXH
